# Locus Coeruleus and Dopamine-Dependent Memory Consolidation

**DOI:** 10.1155/2017/8602690

**Published:** 2017-10-16

**Authors:** Miwako Yamasaki, Tomonori Takeuchi

**Affiliations:** ^1^Department of Anatomy, Hokkaido University Graduate School of Medicine, Sapporo, Hokkaido 060-8638, Japan; ^2^Centre for Discovery Brain Science, Edinburgh Neuroscience, The University of Edinburgh, 1 George Square, Edinburgh EH8 9JZ, UK

## Abstract

Most everyday memories including many episodic-like memories that we may form automatically in the hippocampus (HPC) are forgotten, while some of them are retained for a long time by a memory stabilization process, called initial memory consolidation. Specifically, the retention of everyday memory is enhanced, in humans and animals, when something novel happens shortly before or after the time of encoding. Converging evidence has indicated that dopamine (DA) signaling via D_1_/D_5_ receptors in HPC is required for persistence of synaptic plasticity and memory, thereby playing an important role in the novelty-associated memory enhancement. In this review paper, we aim to provide an overview of the key findings related to D_1_/D_5_ receptor-dependent persistence of synaptic plasticity and memory in HPC, especially focusing on the emerging evidence for a role of the locus coeruleus (LC) in DA-dependent memory consolidation. We then refer to candidate brain areas and circuits that might be responsible for detection and transmission of the environmental novelty signal and molecular and anatomical evidence for the LC-DA system. We also discuss molecular mechanisms that might mediate the environmental novelty-associated memory enhancement, including plasticity-related proteins that are involved in initial memory consolidation processes in HPC.

## 1. Introduction

Many people have vivid memories of the first dinner date with their partner, including details like the name of the restaurant and the food they had. In contrast, it is very difficult to remember what you had for dinner a few weeks ago. Most everyday memories, including episodic-like memories that we may form automatically in the hippocampus (HPC) [1–3], are forgotten, whereas some of them are retained for a long time by a memory stabilization process (initial memory consolidation). Initial selective retention occurs when something novel or salient happens shortly before or after the time of memory encoding, as in “flashbulb memory” [4, 5]. Unexpected novel events create a “halo” of enhanced memory, triggering an initial memory consolidation which extends not only forwards but also backwards in time, boosting retention of trivial memories that would normally be forgotten. Thus, initial consolidation serves as the “gate” to long-term memory, so that only a subset of information is retained for long enough to be subject to stabilization in the neocortex via a complementary process of “systems memory consolidation” [6, 7].

Animal studies of novelty-associated enhancement of memory persistence have enabled analysis of possible mechanisms [8–13] and established that novelty-triggered initial memory consolidation is sensitive to blockade of dopamine (DA) D_1_/D_5_ receptors and protein synthesis inhibitors in HPC. Pharmacological studies of hippocampal synaptic plasticity have supported the notion that D_1_/D_5_ receptors act as a gating mechanism for long-term persistence of plastic changes [14, 15]. However, the literature remains unclear and often contradictory regarding the neuronal source of DA in HPC. An influential hypothesis called the “HPC-VTA (ventral tegmental area) loop” model, proposed over a decade ago [16], postulates that tyrosine hydroxylase- (TH^+^-) expressing neurons of VTA project to the hippocampal formation [17, 18] and release DA under circumstances of novelty or surprise [16, 19]. Nevertheless, VTA-TH^+^ axons are sparse in HPC [17, 18], raising a possibility that other sources of DA, including dense TH^+^ axons from the locus coeruleus (LC), might play a significant role [20, 21].

**Figure 1:**
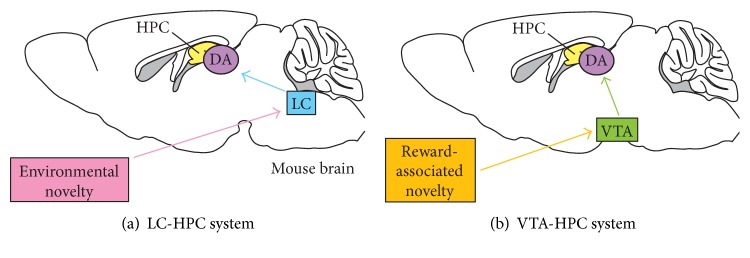
Two distinct novelty systems. There are two types of novelty: “environmental novelty” (e.g., new environment with objects never seen before) and “reward-associated novelty” (e.g., new reward in an unexpected location). They are associated with release of dopamine (DA) in the hippocampus (HPC) but might be processed by different systems with different time windows. (a) The locus coeruleus- (LC-) HPC system mediates environmental novelty which modulates the retention of memory with a broad time window (~1 hr). (b) The ventral tegmental area- (VTA-) HPC system might mediate reward-associated novelty which modulates the memory with a narrow time window.

To seek the neuronal source of hippocampal DA that mediates the beneficial effect of novelty on memory persistence, we combined an optogenetic approach with an everyday memory task in mice. Surprisingly, we found that LC-TH^+^ neurons, originally defined by their canonical noradrenaline (NA) signaling, mediate postencoding novelty-associated enhancement of memory retention in a manner consistent with possible corelease of DA along with NA in HPC [22] (Figure 1(a)). Our results are complemented by the subsequent direct detection of DA corelease from LC axons in HPC [23]. In this review paper, we discuss the following issues with focus on the LC-DA system: (i) a role of hippocampal D_1_/D_5_ receptors in the novelty-induced memory enhancement, (ii) two distinct novelty systems (VTA-HPC and LC-HPC systems) of dopamine-releasing (DAergic) memory modulation, (iii) brain areas that might convey environmental novelty signal to HPC, (iv) molecular and anatomical basis for D_1_/D_5_ receptor-mediated signaling in HPC, and (v) proteins that might mediated the environmental novelty-associated memory enhancement in HPC.

## 2. Novelty-Induced Memory Enhancement Depends on D_1_/D_5_ Receptors in HPC

Activity-dependent hippocampal synaptic plasticity (long-term potentiation (LTP) and long-term depression (LTD)) may underpin the neural mechanisms of hippocampus-dependent learning and memory [3, 13, 24, 25]. Frey and colleagues [26] established the separate existence of early- and late-forms of LTP (E-LTP and L-LTP, resp.) in the hippocampal CA1 region, the latter being defined as protein synthesis dependent. Their work also provided the first experimental evidence suggesting that neuromodulators, especially DA, play a significant role in the transition from E-LTP to L-LTP at CA3–CA1 synapses [27]. DA effects are essentially heterosynaptic rather than homosynaptic (i.e., activity of DAergic inputs affect the strength of other synapses). Hippocampal D_1_/D_5_ receptors play a specific role in control of temporal persistence of LTP at CA3–CA1 synapses ex vivo [12, 14, 15, 28–30]. In awake animals, D_1_/D_5_ receptor activation is crucial for persistence of LTP in CA1, confirming the results ex vivo [10, 28]. Pharmacological manipulations of hippocampal D_1_/D_5_ receptors also indicate that DA is required for the persistence of memories including aversive contextual [31–34], spatial [35, 36], object recognition [33] and paired associate [37] learning. Interestingly, Karunakaran and colleagues showed that learning-induced plasticity of hippocampal parvalbumin neurons was specifically required for long-term memory consolidation through D_1_/D_5_ receptors [38]. Although hippocampal D_1_/D_5_ receptors may play a disproportionate role in the persistence of hippocampal memory, it has also been implicated in facilitating the induction of E-LTP (reviewed in [21]) and, thereby, the entry of information into earlier memory [39].

Since available pharmacological agonists and antagonists of dopamine D_1_-like receptors do not discriminate D_1_ and D_5_ receptors [40], numerous gene knockout studies were conducted in order to elucidate the precise function of D_1_ and D_5_ receptors in roles of hippocampal synaptic plasticity and memory [41–47] (reviewed in [21]). Yet, differentiating the function of hippocampal D_1_ and D_5_ receptors may seem like a daunting task, because there is a caveat in global knockout studies in that they lack regional selectivity. To overcome this issue, Sarinana and colleagues developed knockout mice lacking either D_1_ or D_5_ receptors selectively in granule cells of the dentate gyrus (DG) [48]. They demonstrated that DG-D_1_ receptor deletion, but not DG-D_5_ receptor deletion, impairs persistence of memory in contextual fear conditioning, highlighting the role of DG-D_1_ receptors in gating persistence of hippocampus-dependent memory (but also see [28]). It should be noted, however, that D_5_ receptor mRNA is also expressed strongly in the CA3 and CA1 [48] and LTP at CA3–CA1 synapses ex vivo and spatial memory are impaired in D_5_ receptor global knockout mice [47]. Thus, it is also possible that hippocampal D_5_ receptor outside DG could have an important role in the persistence of hippocampus-dependent memory.

There are many lines of evidence suggesting that the persistence of memory is determined largely by neural activity that occurs at the time of memory encoding. However, the synaptic tagging and capture (STC) hypothesis of protein synthesis-dependent LTP, developed by Frey and Morris [49–51], offers the intriguing but distinct perspective that the persistence of memory is also dependent on independent neural activity afferent to the same pool of neurons mediating synaptic plasticity that occurs before or after memory traces are encoded. According to this hypothesis, the local setting of “synaptic tags” at activated glutamatergic synapses during memory encoding can be dissociated from synthesis and distribution of plasticity-related proteins (PRPs) that is induced by surrounding events (e.g., unexpected novel events). PRPs are then captured by synaptic tags in order to stabilize synaptic changes—a process that is critical for initial memory consolidation.

Indeed, *in vivo* electrophysiological experiments showed that exploration of a novel environment results in facilitation of persistence of synaptic plasticity in the CA1 area [52]. This novelty-associated facilitation of persistence of synaptic plasticity in CA1 was prevented by a D_1_/D_5_ receptor antagonist [10]. Also, considering that exploration of a novel environment leads to upregulation of immediate early genes (IEGs) such as *Arc*/*Arg3.1* and *Homer1a*/*Vesl-1S* [8, 53], the STC hypothesis predicts that unrelated novelty exploration before or after memory encoding should enhance the persistence of a recently encoded memory [3]. This prediction was first confirmed using a hippocampus-dependent inhibitory avoidance task in rats [11]. Our group has developed an “everyday” memory task for rats and mice whose use has revealed that (i) unrelated novel experiences can facilitate the persistence of spatial memory and (ii) this novelty-induced enhancement of memory persistence was prevented by the intrahippocampal injection of a D_1_/D_5_ receptor antagonist (but not by a *β*-adrenoceptor receptor antagonist), or by blockade of hippocampal protein synthesis [12, 13, 22]. Complementary results have been obtained using different learning tasks including inhibitory avoidance, taste memory, object recognition, and contextual fear conditioning [54–58]. Interestingly, Moncada and colleagues showed that novelty-induced memory persistence is also sensitive for hippocampal *β*-adrenoceptor blockade in inhibitory avoidance test [56], in line with *in vivo* electrophysiological results that there are a D_1_/D_5_ receptor-independent mechanism of STC hypothesis [59]. Recently, Nomoto and colleagues elegantly showed that a D_1_/D_5_ receptor-dependent mechanism shared hippocampal neural ensemble for a weak object recognition memory and unrelated novelty is necessary for novelty-induced enhancement of memory persistence [60].

## 3. Two Distinct Novelty Systems of Dopaminergic Memory Modulation in HPC

The prevailing “HPC-VTA loop” model of DAergic consolidation [16] postulates that novelty-associated enhancement of hippocampus-dependent memory is mediated by a subiculum-accumbens-pallidum-VTA-HPC pathway, an idea supported by animal and human studies [32, 61–63]. If this hypothesis holds, then it follows that HPC would receive an innervation from VTA-TH^+^ neurons, environmental novelty would activate VTA-TH^+^ neurons, and activation of VTA-TH^+^ neurons should be necessary and sufficient for novelty-induced enhancement of memory persistence. However, TH^+^ axons from VTA mainly target to the ventral HPC [17, 18, 23, 64, 65] and TH^+^ neurons represent only 10% of hippocampus-projecting neurons in VTA [17], resulting in a sparse projection in the dorsal HPC [22, 23]. Optetrode recordings revealed that VTA-TH^+^ neurons were slightly activated by environmental novelty [22, 66]. Postencoding optogenetic activation of VTA-TH^+^ neurons was without a significant effect on memory persistence. Moreover, pharmacological blockade of VTA-TH^+^ neurons during environmental novelty had no effect on novelty-associated memory enhancement [22]. Importantly, the impact of “environmental novelty” may differ qualitatively from that of “reward-associated novelty.” Reward expectancy is a critical component of the execution of learned actions until they become habitual [67]. Longstanding data point that the substantia nigra (SN)/VTA system thought to play important role for processing unexpected reward [68–70]. Such reward signals are primarily coded by DA, which modulates the synaptic connections in the striatum within a narrow time window [71]. Considering that memory retention is also enhanced by reward magnitude [12, 22, 72], we now hypothesize that VTA-HPC system might mediate reward-associated novelty which modulates the retention of memory with a narrow time window (Figure 1(b)). Keeping with this hypothesis, there was a narrow time window for impact of pharmacological VTA inactivation on both synaptic plasticity *in vivo* and memory in the passive avoidance task [73]. Optogenetic activation of hippocampus-projecting VTA-TH^+^ axons can bidirectionally modulate CA3–CA1 synaptic responses ex vivo [74], and optogenetic activation of VTA-TH^+^ axons in HPC at the time of learning enhances spatial memory after 1 hr [66]. Interestingly, VTA activation associated with visual novelty did not correlate with memory enhancement in humans [75]. In contrast, recent study in humans have demonstrated that postlearning SN/VTA-hippocampal interactions contribute to preferential retention of episodic memory that are learned in high-reward contexts [76].

**Figure 2:**
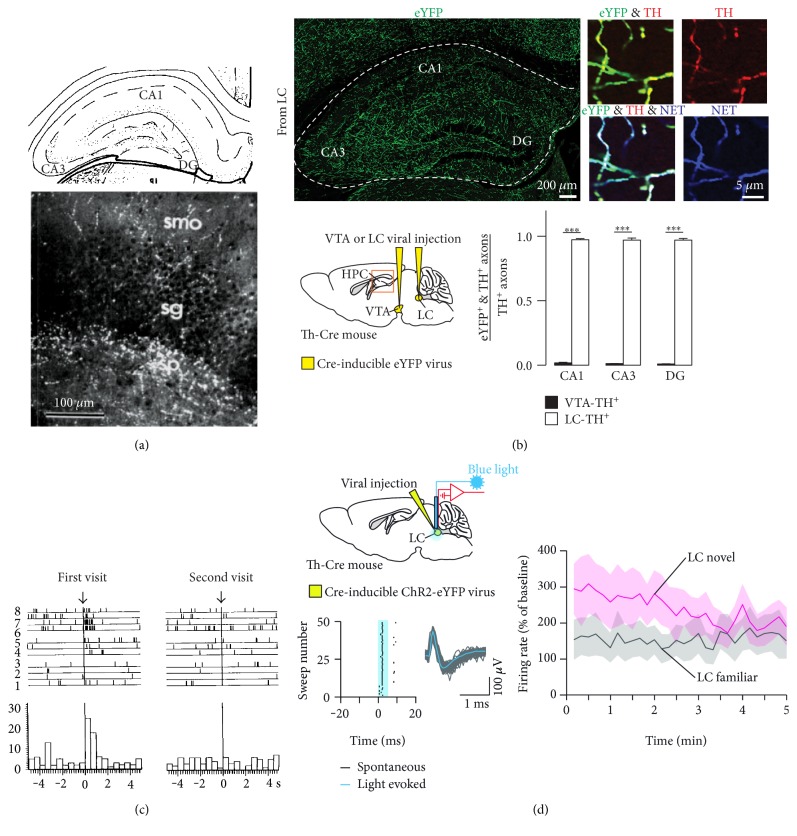
Hippocampal projections from LC neurons and increased LC neuron activity by environmental novelty. (a) Immunofluorescence of D*β*H in HPC. (a) is reproduced from [88]. (b) TH^+^ axons in the dorsal HPC originate from LC-TH^+^ neurons. Quantification shows stronger TH^+^ projections from LC than from VTA in CA1, CA3, and DG. ^∗∗∗^*p* < 0.001 , paired t-test. (b) is reproduced from [22]. (c) Response to novelty and its habituation in LC neurons. (c) is reproduced from [96]. (d) LC-TH^+^ neurons show strong response to environmental novelty that habituates over 5 min. (d) is reproduced from [22].

Considering that DA acts not only as a neurotransmitter in its own right but also as the precursor for NA, TH^+^ axons originating from the LC (A6, in rat nomenclature) [77] are another potential source of DA in HPC. The LC has long been implicated in novelty, attention, arousal, and cognition [78–83], and its firing is tied to distinct changes in neocortical activation during sleep [84]. The LC receives prominent direct inputs from many cortical and subcortical areas and sends extensive projections throughout the brain and spinal cord with the exception of the basal ganglia and SN, all of which are dense with axonal projections or cell bodies of DAergic SN/VTA neurons [85, 86]. Dense innervation of all hippocampal areas by LC axons has been demonstrated by prior anatomical studies (Figure 2(a)) [87–93]. Recently, cell type-specific tract tracing experiments have confirmed these observations and further established that TH^+^ axons from LC far outnumber those from VTA (Figure 2(b)) [22, 23]. The LC has two different types of firing patterns: constant “tonic” activity (1–3 Hz) and intermittent “phasic” impulse activity (8–10 Hz) [78], that have been correlated to different behavioural states [94]. The LC neurons are activated in response to environmental novelty that habituates over time (Figures 2(c) and 2(d)) [22, 95, 96].

**Figure 3:**
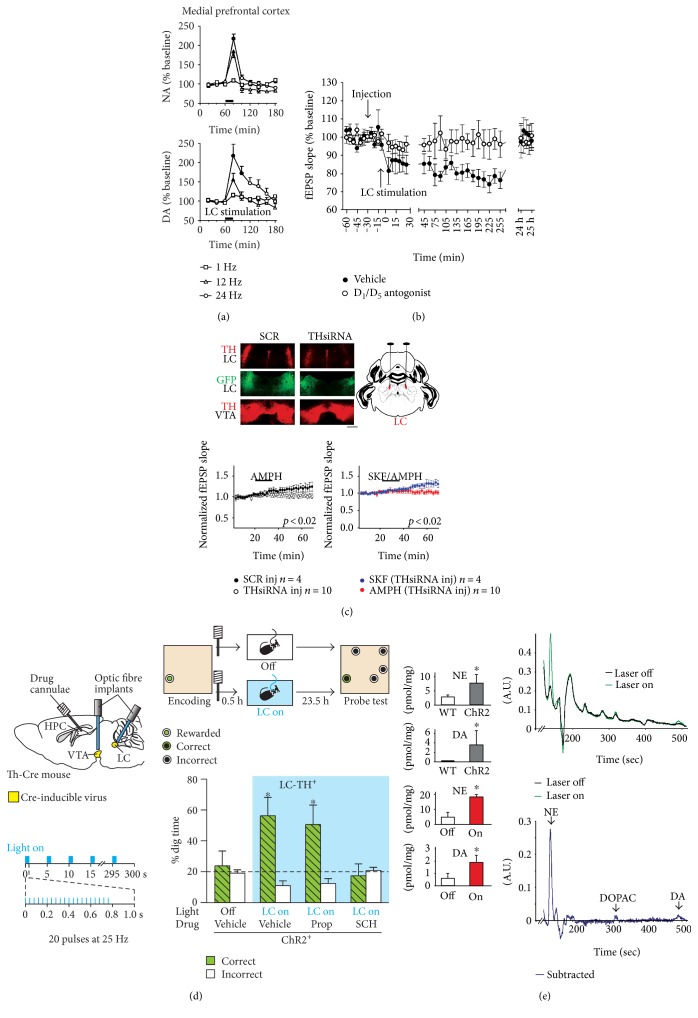
Noncanonical release of DA from LC-TH^+^ axons in HPC. (a) LC electorical stimulation-induced increase of NA (top) and DA (bottom) in the medial prefrontal cortex. (a) is reproduced from [106]. (b) LC electorical stimulation-mediated D_1_/D_5_ receptor-sensitive facilitation of CA3–CA1 LTD *in vivo*. (b) is reproduced from [52]. (c) TH knockdown in LC prevents D_1_/D_5_ receptor-mediated enhancement of excitatory transmission in HPC. (c) is reproduced from [20]. (d) Optogenetic activation of LC-TH^+^ neurons enhances persistence of memory in a manner consistent with release of DA in HPC ^∗^*p* < 0.05 versus chance, t-test. (d) is reproduced from [22]. (e) Optogenetic activation of LC-TH^+^ axons in HPC produces an increase in DA release in the dorsal HPC. ^∗^*p* < 0.05, t-test. (e) is reproduced from [23].

Pharmacological inhibition of LC prevents the beneficial effect of environmental novelty on memory persistence [22]. Critically, postencoding optogenetic activation of LC-TH^+^ neurons mimics this environmental novelty effect (Figure 3(d)). Surprisingly, this LC-TH^+^ neuron photoactivation-driven memory enhancement is sensitive to hippocampal D_1_/D_5_ receptor blockade and resistant to *β*-adrenoceptor blockade (Figure 3(d)). In line with these results, electrical activation of LC results in persistent synaptic plasticity at CA3–CA1 synapses *in vivo*, which is prevented by D_1_/D_5_ receptor antagonist (Figure 3(b)) [52]. Furthermore, selective optogenetic activation of hippocampus-projecting LC-TH^+^ axons mediates a D_1_/D_5_ receptor-sensitive and *β*-adrenoceptor-resistant enhancement of synaptic transmission and LTP at CA3–CA1 synapses ex vivo [22], consistent with the idea that LC-TH^+^ might release DA in HPC [20, 97]. Our results are complemented by the subsequent direct detection of DA corelease along with NA from LC-TH^+^ axons in HPC (Figure 3(e)) [23]. Taken together, these observations collectively indicate that LC-HPC system is activated by environmental novelty and mediates postencoding memory enhancement via the noncanonical release of DA in HPC (Figure 1(a)).

In contrast, a recent study showed that electrical activation of LC can mimic the beneficial effect of environmental novelty on memory persistence of the inhibitory avoidance and spatial object recognition tasks in rats in a hippocampal *β*-adrenoceptor-sensitive manner [61]. Further studies will be required to access how the DAergic and noradrenergic systems interact mechanistically in processing environmental novelty in HPC.

It is not yet clear, however, how the environmental novelty signal reaches the LC-TH^+^ neurons. Computational models [98] have proposed that novelty is computed in the hippocampal CA1 through a process that compares the “predictions” that arrive from CA3 via the Schaffer collaterals with the “reality” that arrives directly from the neocortex via the perforant path. According to this view, CA1 acts as a “comparator” that detects mismatches between predictions from CA3 and actual sensory input from the neocortex [16]. Based on this model, one possibility is that novelty detection occurs in HPC, which then activates LC-TH^+^ neurons that project back to HPC. There has been, however, little direct empirical evidence to support the CA1 comparator model so far. In addition, a recent study [86] found no direct projections from HPC to LC-TH^+^ neurons. Therefore, it is likely that the environmental novelty signal reaches LC-TH^+^ neurons from HPC via a relay (e.g., the medial prefrontal cortex [99]). Second possibility is that LC-HPC projection is part of a parallel circuit independent of the HPC-VTA loop. There are many areas of the brain that will respond stronger to novel stimuli. Among them, the superior colliculus shows strong response to novel visual stimuli [100] as well as novel multisensory information [101]. Neurons in the superior colliculus habituate their novelty response over time in a similar way to the environmental novelty-associated response in LC neurons. It is also noted that the superior colliculus constitutes a large fraction of direct synaptic input to LC-TH^+^ neurons [86].

## 4. Molecular and Anatomical Basis for D_1_/D_5_ Receptor-Mediated Signaling in HPC

In catecholamine synthesis pathway, TH is the rate-limiting enzyme under basal conditions. However, when D*β*H (dopamine-*β*-hydroxylase), the enzyme that converts DA to NA in synaptic vesicles of LC-TH^+^ terminals, becomes saturated and rate limiting [102, 103], not all of the DA in the vesicle is converted to NA, and the probability of corelease of DA and NA would increase. In support of this hypothesis, it has been demonstrated that chemical and electrical stimulation of LC neurons elicits release of both DA and NA in the medial prefrontal cortex (Figure 3(a)) [97, 104–106] and HPC [107, 108]. Smith and Greene were the first to provide direct electrophysiological evidence for this idea (Figure 3(c)) [20]. More recent optogenetic studies have further provided physiological and biochemical evidence for noncanonical release of DA from LC-TH^+^ axons in HPC (Figures 3(d) and 3(e)) [22, 23]. Taken together, it is thus plausible that LC-TH^+^ axons are the source of DA in the dorsal HPC.

In DA signaling, dopamine transporter- (DAT-) mediated reuptake plays a key role in limiting DA diffusion and defining DA transients [109]. Similar to the sparse expression in the medial prefrontal cortex [110, 111], however, DAT expression is extremely low in HPC [112–114]. Instead, norepinephrine transporter (NET), which also has an affinity for DA [97, 115, 116], is abundantly expressed on the plasma membrane of LC-TH^+^ axons in HPC. As is the case for the medial prefrontal cortex [117], heterologous reuptake by NET contributes to the clearance of DA in HPC [118, 119]. Although the difference between the kinetics and efficacy of DA reuptake by DAT and NET remains elusive, the major DA clearance system in HPC is similar to the medial prefrontal cortex, where slow and sustained pattern of DA release is observed during a large variety of cognitive and motivational functions [120].

Now that it has been established that LC-TH^+^ axons are likely to be an essential constituent of DA signaling in the dorsal HPC, it is imperative to further explore their distribution patterns and as well as their connectivity with hippocampal principal neurons and various types of interneurons. As consistently demonstrated in prior studies by D*β*H immunohistochemistry as well as autoradiography [88, 89, 91, 93], there are some regional and laminar differences in innervation density of LC axons. To summarize simply, LC innervation covers the entire HPC, and it is especially high in DG. Laminar distribution pattern is also different depending on subregions. In the subiculum and CA1, the density of LC axons is clearly higher in the stratum lacunosum moleculare. In CA3, the highest density is found in the stratum lucidum, where mossy fibers of DG granule cells make synapses on pyramidal neurons. In DG, it is the highest in the polymorph layer in the hilus and the lowest in the granule cell layer (but see [23]). It should be also noted that the density of LC axon is moderately high in the molecular layer. Thus, the differential distribution pattern within each region suggests that the cellular targets of LC-TH^+^ axons might differ depending on the subregions. Furthermore, considering that different subregions exercise distinct functions in information processing within HPC [121], it would be noteworthy that the densest regional LC-TH^+^ innervations in HPC are those of the DG and subiculum, which correspond to its main cortical input and output stations, respectively [122, 123].

Of further consideration is whether specialized DA release sites exist on LC-TH^+^ axons, and if so, how these DA release sites are distributed in HPC, especially in relation to localisation of D_1_ and D_5_ receptors. In this regard, we are still at the very beginning of the path to get the whole picture. For example, the synaptic profile of TH^+^ axons in HPC is still a controversial issue. Previous immunoelectron microscopic analyses have shown that TH^+^ axons often make direct contact with pyramidal neurons and *γ*-aminobutyric acid-releasing (GABAergic) interneurons [90, 124, 125]. Even at such contact sites, however, the great majority of them do not form synapse-like specializations, including uniform cleft width between the apposed membranes and thickening of the apposed membranes [90, 125, 126]. By contrast, a small fraction of them seem to make symmetrical synapses with soma and dendritic shaft of GABAergic interneurons [90]. In recent years, however, it has become clear that morphologically defined “DA synapse,” which is formed between TH^+^ terminals and dendritic elements that exhibit ultrastructural features of symmetrical synapses, is not likely to be the site of DA transmission. Specifically, D_1_ receptors are almost exclusively located at the extrasynaptic membrane [127, 128] and not localized to DA synapses [129]. Thus, future studies are required to determine the release site of DA in LC-TH^+^ axons and their spatial relationship with D_1_ and D_5_ receptors in HPC.

Our current knowledge regarding the expression pattern of D_1_ and D_5_ receptors in HPC is still limited and inconclusive [48, 130–138]. Distribution of D_1_/D_5_ receptors in HPC was first demonstrated by binding studies using radiolabelled ligands. Although the signal intensity in HPC is much lower than in “DA-rich regions” such as the striatum, low to moderate levels of binding to D_1_/D_5_ receptors are observed in the molecular layer of DG [130, 139–142]. In situ hybridization studies have further uncovered differential expression patterns of D_1_ receptor mRNA in the ventral and dorsal HPC. D_1_ receptor mRNA is expressed in dispersed cells in CA3/CA1 and DG in the ventral HPC, while it is mainly expressed in DG granule cells in the dorsal HPC [48, 130, 142]. These observations are further supported by a recent study on transgenic mice expressing eGFP (enhanced green fluorescent protein) under control of the D_1_ receptor promotor, which shows that it is mainly expressed in DG granule cells and a subset of GABAergic interneurons in the hilus and CA1/CA3 [137, 138]. In spite of this clear expression pattern, subcellular distribution of D_1_ receptor remains elusive, mainly because D_1_ receptor protein expression in HPC is quite low compared with the striatum. In situ hybridization studies have consistently shown that D_5_ receptor mRNA is dominantly expressed in HPC [48, 131–133]. At the cellular level, there is a consensus that D_5_ receptor is expressed in pyramidal neurons in CA1/CA3 and granule cells in DG [48, 131–134]. However, further analyses are needed in order to determine its subcellular localization and expression in GABAergic interneurons.

It is now widely accepted that DA receptors can form both homomers and heteromers with several other classes of receptors, including other G protein-coupled receptors (GPCRs) and ionotropic receptors [143, 144]. D_1_ receptor directly couples with the GluN1 and GluN2A subunits of the *N*-methyl-D-aspartate (NMDA) receptor and modulates the NMDA receptor currents [145, 146]. Recently, Kern and colleagues showed that D_1_ receptor and ghrelin receptor form heteromers in a complex with G*α*q and initiate a noncanonical cAMP-independent signaling pathway that regulate DA-dependent hippocampal synaptic plasticity and memory [147]. Similarly, D_5_ receptor directly couples to the *γ*2 subunit of the GABA subtype-A receptor, modulating the inhibitory current [148].

## 5. Plasticity-Related Proteins and Novelty-Associated Memory Enhancement in HPC

Optogenetic activation of hippocampus-projecting LC-TH^+^ axons at the time of learning enhances a D_1_/D_5_ receptor-sensitive 24 hr memory in a spatial object recognition task [23]. However, from the perspective of the STC hypothesis [49, 51], our behavioural protocol [22], in which there is a 30 min delay between encoding and exposure to environmental novelty, can dissociate the encoding phase from the consolidation processes. It could allow us to exclude the possibility of DAergic modulation of memory encoding via, for example, changes in attention [23, 149] and alterations in CREB- (cyclic adenosine monophosphate response element-binding protein-) mediated changes in neuronal excitability [150]. Our proposed mechanism for postencoding environmental novelty-associated memory enhancement is as follows: hippocampal D_1_/D_5_ receptor activation induced by environmental novelty triggers nuclear gene transcription and nuclear/dendritic synthesis and distribution of PRPs that are captured by “synaptic tags” in order to stabilize synaptic changes within hippocampal excitatory neurons [51].

Pharmacological activation of D_1_/D_5_ receptors enhances Zif268/Egr-1/Krox-24 and Arc expression in DG *in vivo* [151]. D_1_/D_5_ receptor activation also stimulates local protein synthesis in the dendrites of hippocampal neuron *in vitro* [152, 153]. On the other hand, LTP-induced expression of Zif268 and Arc in CA1 is significantly reduced in global D_1_ receptor knockout mice [44, 46]. It has been established that exploration of a novel environment causes upregulation of several IEGs in HPC [8, 154–156]. However, important questions remain open regarding the specific role of particular PRPs in novelty-induced enhancement of memory persistence. Although several proteins, including Homer1a, Arc, BDNF (brain-derived neurotrophic factor), AMPA (*α*-amino-3-hydroxy-5-methyl-4-isoxazole propionate) receptor, actin and PKM*ζ* (protein kinase M*ζ*), have been suggested as possible key mediators of persistence of long-lasting synaptic plasticity and memory [157], they only provide partial explanations of the phenomenon. For example, synaptic activity-induced *Homer1a* and *Arc* gene products are targeted to active or inactive synapses, respectively, *in vitro* [158, 159], but their roles in environmental novelty-induced memory persistence remain largely unexplored.

The local setting of synaptic tags and the capture of PRPs by tagged synapses might have occurred in activated dendritic spines at glutamatergic synapses in HPC. The capture of PRPs by tagged synapses, critical for initial memory consolidation, results in an increase of both the strength of the synaptic transmission (“functional plasticity”) and volume of dendritic spines (“structural plasticity”) [51]. Functional and structural plasticity is thought to involve the insertion of AMPA receptors at the postsynaptic membrane [160] and the remodelling of actin cytoskeleton [161, 162], respectively. Thus, we predict the features of PRPs to be as follows: PRPs are (i) enriched in dendritic spines and (ii) involved in the regulation of AMPA receptor trafficking and/or remodelling of actin cytoskeleton. It has been reported that 1755 gene products are enriched in postsynaptic dendritic spines (SynaptomeDB, http://metamoodics.org/SynaptomeDB/index.php [163]).

**Figure 4:**
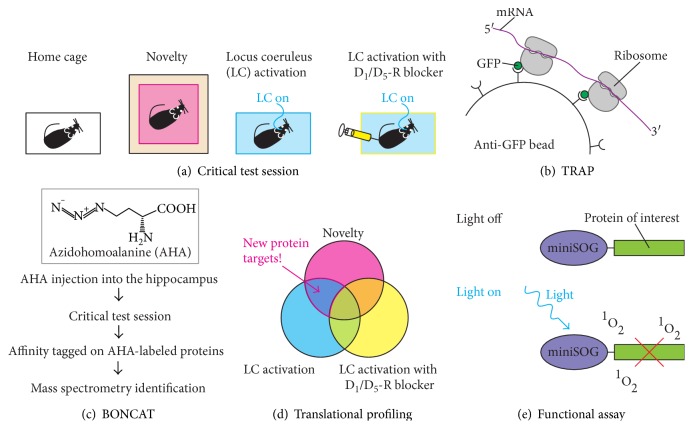
Identification of key PRPs (plasticity-related proteins) by using optogenetics and translational profiling. (a) The critical test session would include (i) a behavioural condition that enhances memory (novelty), (ii) optogenetic activation of LC neurons (LC on), and (iii) LC activation with D_1_/D_5_ receptor blocker (LC on with D_1_/D_5_-R blocker) that might block the relevant synthesis of PRPs mediated by DAergic signaling in key target neurons. These conditions are compared to a home cage condition. (b) The TRAP technology, involving cell type-specific expression of green fluorescent protein- (GFP-) tagged ribosomal protein and GFP immunoprecipitation, enables the selective isolation of “translated mRNAs” in genetically defined neurons. (c) BONCAT (bioorthogonal noncanonical amino acid tagging) technology, involving labelling of newly synthesized proteins by AHA (azidohomoalanine), which can be later tagged for isolation and identification by mass spectrometry. (d) Candidate PRPs would be identified through the Venn diagram overlap of experimental conditions. (e) Optogenetic inhibition of a candidate PRP using “miniSOG,” a genetically encoded singlet oxygen generator [168]. After light illumination, singlet oxygen (^1^O_2_) is generated by miniSOG leading to the inactivation of fusion protein of interest.

One possible experiment for identifying key PRPs critical for environmental novelty-induced memory boost would be translational profiling acquired under different behavioural and physiological conditions (Figure 4). The intellectual background to this approach is STC hypothesis [49, 51] whereby the mechanisms mediating memory encoding (tag-setting) and consolidation (sequestration of PRPs) are independent events. Previous results [164] support this dissociation between tag-setting (calcium/calmodulin-dependent protein kinase (CaMK) II signaling pathway) and the availability of PRPs (CaMKIV signaling pathway). The critical test session after which tissue is taken would include novelty exploration and optogenetic activation of LC-TH^+^ neurons that can enhance memory retention (Figure 4(a)) [22]. In addition, it would include photoactivation of LC-TH^+^ neurons with systemic injection of D_1_/D_5_ receptor antagonist that might block the relevant synthesis of PRPs mediated by DAergic signaling in hippocampal neurons. These conditions would be compared to a baseline home cage condition. Recently developed techniques “TRAP” (translating ribosome affinity purification) (Figure 4(b)) [165] and “BONCAT” (bioorthogonal noncanonical amino acid tagging) (Figure 4(c)) [153] allow us to selectively isolate translated mRNAs and newly synthesized proteins during the critical test session, respectively. Translational profiles acquired under different behavioural and physiological conditions would be then compared (Figure 4(d)). Specifically, comparisons among a subset of genes translated in these different conditions can be used to zero-in on candidate PRPs.

If candidate PRPs would be identified, the next logical step is to assess whether the candidate PRPs are preferentially targeted to activated spines using two-photon glutamate uncaging with time-lapse imaging [166]. Subsequently, it is imperative to characterise the function of the candidate PRPs that are induced by environmental novelty in novelty-associated enhancement of memory persistence. Methods to optically control the activity of specific proteins [167], when available, would allow us to disable the function of the candidate PRPs by illumination with light during initial memory consolidation in a spatially and temporally precise manner (Figure 4(e)). These sets of experiments would identify key PRPs that mediate novelty-associated enhancement of memory persistence within excitatory neurons in HPC. Among the brain disorders, the breakdown of memory (associated with stress, aging, and age-associated disorders) causes great concern. Identification of proteins that enhance retention of everyday memory will have the potential to reveal new drug targets for treatment or restoration of lost memory function. These proteins will also constitute good candidates for “biomarkers” for impairments such as forgetfulness and age-associated memory decline.

## 6. Conclusions

Most everyday memories may form automatically in HPC. The key role of this memory system is to filter our unnecessary information but keep the important memories by a mechanism that involves novelty-associated DA release in HPC. Recent optogenetic studies have revealed that projections from noradrenergic LC-TH^+^ neurons to HPC can drive the postencoding environmental novelty-associated enhancement of memory retention through noncanonical release of DA in HPC. These studies also raise an intriguing possibility that the impact of environmental novelty may differ qualitatively from that of reward-associated novelty and projections from VTA-TH^+^ neurons to HPC might mediate reward-associated novelty which modulates the memory retention with a narrow time window. Initial consolidation triggered by two distinct dopaminergic novelty systems could help make encoded memory traces last long enough for the effective function of the more extended process of system consolidation by which hippocampus-dependent memories guide the eventual stabilization of neocortical memory networks.
